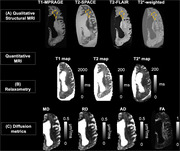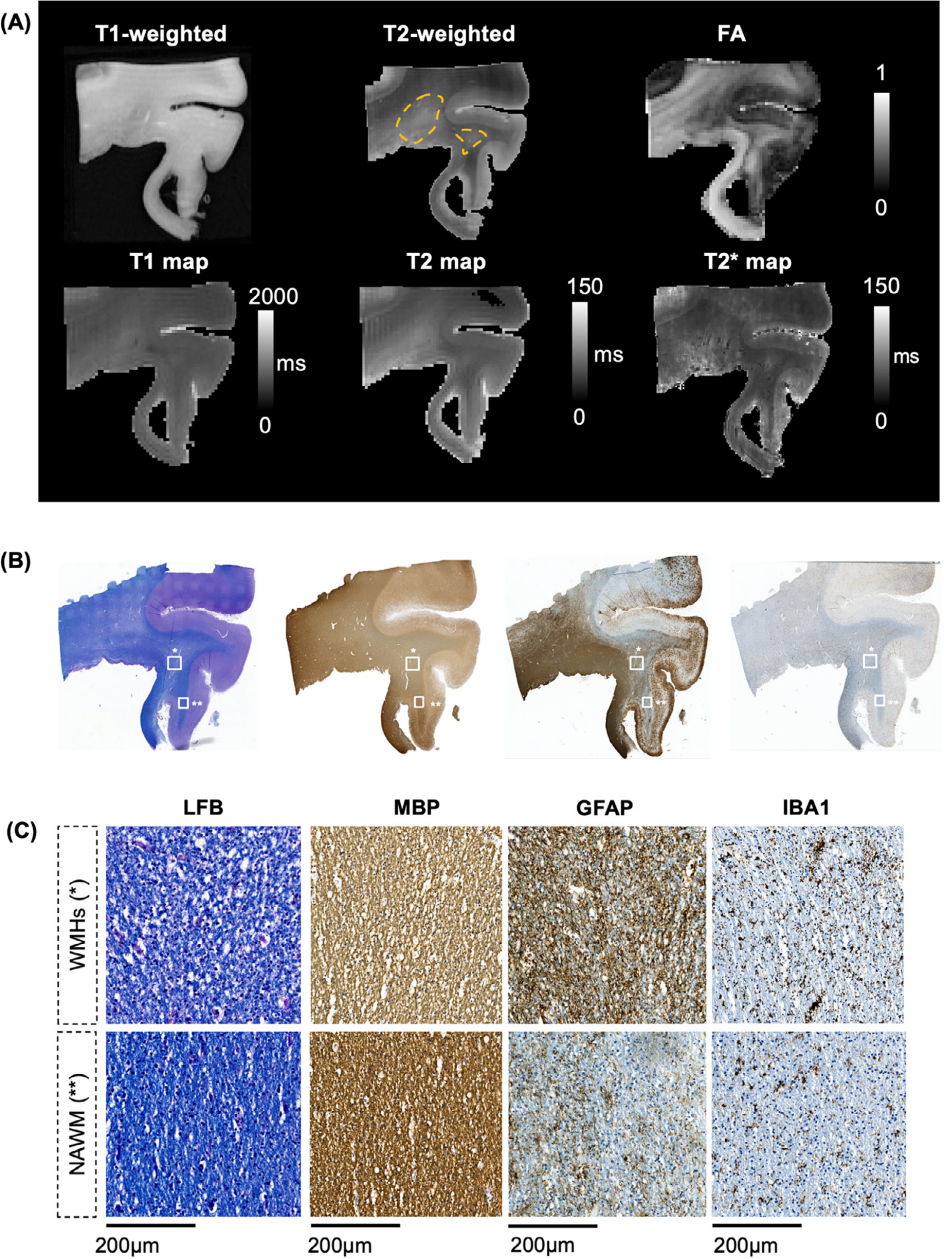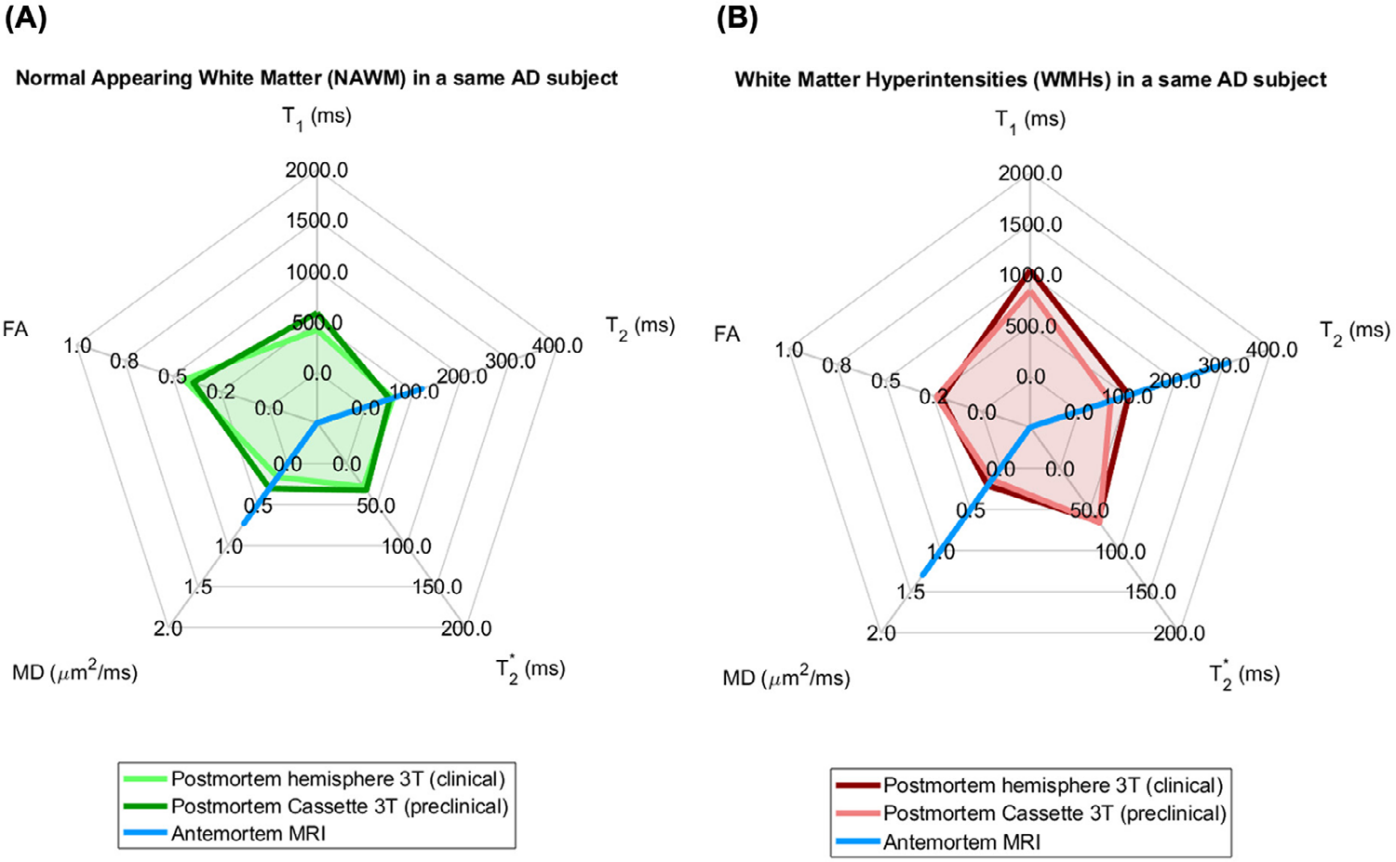# Multiparametric Postmortem MRI and Histopathological Analysis Reveals Complex Signal Origins of White Matter Hyperintensity in Alzheimer's Disease

**DOI:** 10.1002/alz70855_102276

**Published:** 2025-12-23

**Authors:** Chenyang Li, Dominique Leitner, Huize Pang, Mary Bruno, Thomas Wisniewski, Arline Faustin, Youssef Zaim‐Wadghiri, Jiangyang Zhang, Yulin Ge

**Affiliations:** ^1^ NYU Grossman School of Medicine, New York, NY, USA; ^2^ Center for Cognitive Neurology, New York University Langone Health, New York, NY, USA; ^3^ Alzheimer's Disease Research Center, New York University Langone Health, New York, NY, USA; ^4^ New York University Grossman School of Medicine, New York, NY, USA

## Abstract

**Background:**

White matter hyperintensities (WMHs), visible as increased signal intensity on T2‐FLAIR MRI images, represents a key imaging biomarker in the Alzheimer's Disease and related dementias (AD/ADRD). Histopathology links WMHs to demyelination, axonal degeneration and loss, reactive astrogliosis and microglial activation. However, direct quantitative correlations between *in vivo* MRI signals and specific histopathological features are challenging due to differences in spatial resolution and the indirect nature of MRI. This study aims to bridge clinical MRI findings of WMHs with histopathological findings using multiparametric and multiscale MRI approaches in dementia brains.

**Method:**

Two post‐mortem brain specimens from pathologically confirmed AD cases were included in this study. The imaging protocol includes sequential MRI acquisitions at 3T. Whole‐hemispheric imaging was initially performed using a 3T clinical system. The specimens were then sectioned into tissue blocks sized to fit histological cassettes. For high‐resolution ex vivo imaging, each tissue block was imaged on a preclinical 3T MRI system, using the same imaging protocols as the hemispheric scans but with improved resolution.

**Result:**

Postmortem hemisphere scans on a clinical scanner demonstrated that WMHs showed comparable contrast to in vivo imaging. Quantitative analysis of multimodal imaging data of WMHs from both preclinical and clinical 3T scanners, exhibited increased mean diffusivity (MD) and prolonged T1, T2, and T2* relaxation times compared to surrounding normal appearing white matter (NAWM). Histopathological staining confirmed spatial overlap between tissue block MRI and histological findings of WMHs, highlighting pathological features of white mater vacuolation, demyelination, astrogliosis and neuroinflammation.

**Conclusion:**

In conclusion, the joint analysis of high‐resolution postmortem MRI and histopathology revealed a complex signal origin of WMHs, characterized by elevated diffusivity and prolonged T1 and T2 relaxation times, which correlate with various histological findings. This preliminary study may provide insights into the signal composition of WMHs *in vivo*.

**Figure 1**. Representative half hemisphere MRI data used to evaluate WMHs.

**Figure 2**. High‐resolution MRI of small tissue cassettes at 3T and (B) Histology of WMHs using LFB, MBP, GFAP, and IBA1 staining of WMHs.

**Figure 3**. Spider plot of the multiparametric measurements between (**A**) NAWM and (**B**) WMHs from a single subject.